# Evaluating the Effect of Using Training CDs on the Patients with Type II Diabetes

**Published:** 2018-02

**Authors:** Hamid TAGHINEJAD, Hamed TAVAN

**Affiliations:** 1. Dept. of Nursing, Faculty of Nursing and Midwifery, Ilam University of Medical Sciences, Ilam, Iran; 2. Dept. of Nursing, Student Research Committee, Ilam University of Medical Sciences, Ilam, Iran

## Dear Editor-in-Chief

With a variety of short-term and long-term complications, diabetes is one of the most common diseases in the world and has negative effects on different body systems (e.g., brain, heart, kidneys) (1). The prevalence of diabetes is high in Iran and one per 20 of the Iranian people suffers from diabetes. However, the most important problem is that most people do not know they are sick and this causes irreparable consequences (1).

Although diabetes is a common disease, some provisions can be thought to prevent its progress and in some cases prevent its occurrence. One of these cases is training diabetic patients. Training can be done through different ways such as using pamphlets, leaflets, photographs, videos, and training classes about the disease for patients. With regard to the evolution of technology and new instruments, better and more effective methods are implemented for training in the world (2, 3).

In this study, patients with diabetes were trained through instructional videos. First, patients with type II diabetes referred to the clinic were identified. Then, the date and time of their next visit to the clinic were arranged. Moreover, the objectives of the study were explained to the participants. The samples were divided into two groups: case and control groups. Initially, the two groups were given a test battery including demographic information (i.e., age, gender, height, weight, marital status, location, and history of disease) and 10 questions on their knowledge and attitudes about diabetes, in which the 5-point Likert-type scale was used (Completely know = 5, Partly know = 4, Not sure = 3, Slightly know = 2, and Do not know = 1). Overall, the total score of the questionnaire was 50 points and it was classified into four categories including the low score category (10–20 points), the lower-middle score category (21–30 points), the upper-middle score category (31–40 points), and high score category (41–50 points). The sample size based on the following statistical formula was at least 44 patients in each group:

$$n=\frac{{\left(Z1+Z2\right)}^{2}{\left(2S\right)}^{2}}{d^{2}}$$

*Z1* is a 95% confidence interval (i.e., 1/96). *Z2* is the power of a test (0/84 for 80% power).

*S* is an estimate of the standard deviation of the health literacy score of the two groups. *d* is the minimum mean difference between the two groups and shows the difference is significant while *S* is considered to be 0/6.

The questionnaire was completed by the case and control groups. Only the case group was given the training CDs containing information on recognizing diabetes, its causes and complications, diet, physical activities and other methods to control diabetes, and required actions at the onset of diabetes. After going to the clinic again, the patients completed the same questionnaire. Furthermore, the control group also completed the same questionnaire on their next visit to the clinic. Before the intervention (i.e., the instructional videos), the scores of the two groups were almost similar and close to each other. In terms of classifications, scores of the case group were as follows: 68% had low score, 24% received the lower-middle score, and 8% achieved the upper-middle high score, respectively. In the control group, scores were classified as follows: 65% had low score, 25% received the lower-middle score, and 10% achieved the upper-middle score.

Moreover, the mean and standard deviation of the case group was 8.6 ± 21.7 and those of the control group were 8.69 ± 22.3. The scores of both groups had increased after the intervention. Although the control group experienced no training, their scores were higher due to conducting the instructions of the experts. However, their scores were not statistically significant (*P*-value=0.03). However, the training (with instructional videos) was carried out for the case group and their scores were higher in the next test and statistically significant (*P*-value = 0.03).

After the second visit, the classification of scores of both groups was as follows: in the case group, 34% had low score, 46% received the lower-middle score, and 20% achieved the upper-middle score and in the control group, 25% had low score, 55% received the lower-middle score, and 20% achieved the upper-middle score. The mean and standard deviation of the case and control groups were10.9 ± 27.5 and 9.8±.33.8, respectively. However, the striking thing is that the overall information on type II diabetes were presented to the patients, but none of them achieved high scores (41–50 points). One of the reasons is that the patients did not exactly pay attention to the questions while responding or to the instructional videos while watching. Most of the study participants were people over 50 yr and maybe this is one of the reasons that the training was not done efficiently.

Finally, if some training for effective prevention or treatment of the disease is conducted through instructional videos, it will bring about better results for patients. Thus, it is necessary for the practitioners to act with sensitivity about this subject for example by preparing appropriate programs and lectures or making some series about diabetes on the national radio and television broadcasts to grant facts because the intuitive teaching method might be much more fruitful than other training methods.
